# Predictive Maintenance of Boiler Feed Water Pumps Using SCADA Data

**DOI:** 10.3390/s20020571

**Published:** 2020-01-20

**Authors:** Marek Moleda, Alina Momot, Dariusz Mrozek

**Affiliations:** 1 TAURON Wytwarzanie S.A., Promienna 51, 43-603 Jaworzno, Poland; 2 Department of Applied Informatics, Silesian University of Technology, Akademicka 16, 44-100 Gliwice, Poland

**Keywords:** predictive maintenance, Internet of Things, boiler feed pump, SCADA, anomaly detection

## Abstract

IoT enabled predictive maintenance allows companies in the energy sector to identify potential problems in the production devices far before the failure occurs. In this paper, we propose a method for early detection of faults in boiler feed pumps using existing measurements currently captured by control devices. In the experimental part, we work on real measurement data and events from a coal fired power plant. The main research objective is to implement a model that detects deviations from the normal operation state based on regression and to check which events or failures can be detected by it. The presented technique allows the creation of a predictive system working on the basis of the available data with a minimal requirement of expert knowledge, in particular the knowledge related to the categorization of failures and the exact time of their occurrence, which is sometimes difficult to identify. The paper shows that with modern technologies, such as the Internet of Things, big data, and cloud computing, it is possible to integrate automation systems, designed in the past only to control the production process, with IT systems that make all processes more efficient through the use of advanced analytic tools.

## 1. Introduction

Electricity production is a type of continuous manufacturing process where, for economic reasons, devices are often serviced, which results in a low number of major failures. On the other hand, some component units often operate in a state of failure in order to maintain the production process. Therefore, when analyzing process data, we have to face the challenge of correct interpretation of that data. The priority is to maintain production capacity by keeping equipment in good condition. Among the devices that have a critical impact on electricity production in power plants are boiler feed pumps. The failure of these devices may cause the whole unit to cease production. Therefore, it is justified to cover them with the condition monitoring methodology [1]. The condition monitoring gives us the opportunity of using the predictive maintenance (rather than “fix when fail” approach) applying industrial IoT services [2]. In this work, we will also try to apply condition monitoring based on data from SCADA systems (Supervisory Control And Data Acquisition) [3].

Owing to equipment monitoring and fault detection, it is possible to make a transition from preventive to predictive maintenance methods. Predictive maintenance requires comprehensive information on the current state of health of the devices. Additional metering, physical process modeling, or data driven models can be used to obtain the information. The integration of systems and the possibility of using advanced analytics on data from the existing systems allowed for the rapid development of data driven methods in this area, providing opportunities to gain insight in an inexpensive way. Predictive maintenance provides better scheduled fixing plans, minimizing planned and unplanned downtimes. It means [4]:

- reducing unnecessary repairs of equipment in good condition,
- minimizing the probability of downtime by real-time health monitoring,
- better asset management by estimating the remaining useful life.

In reference to the proposal presented in the article [2], treating the existing SCADA metering and its data repository as an industrial Internet of Things environment, we will create an IoT service to increase the availability of the devices by using predictive maintenance techniques. By using cloud computing [5,6,7] and direct access to operational data, we can create new value at a low cost for operators and engineers managing the production process.

A significant problem with analytical work in the production environment is to determine the veracity of the data [8,9,10]. In the case of manually entered data, such as data entered in failure logs, the data rarely contain the specific time of the event, and the content requires interpretation by an expert. This poses challenges to correct categorization of events and correct timing of their occurrence in the time series of data streams coming from sensors. Moreover, apart from the problem of measurement error, sensor data are often subjected to interference from other devices. For example, the measured vibration value may come from the connected component; temperature indications are significantly influenced by weather conditions. Sometimes, the recorded measurement values do not come from sensors, but are simulated values. This practice is used to avoid false alarms in security systems. The above aspects significantly differentiate the production environment from the laboratory environment, forcing the data to be treated with a high degree of uncertainty.

**Figure 1:**
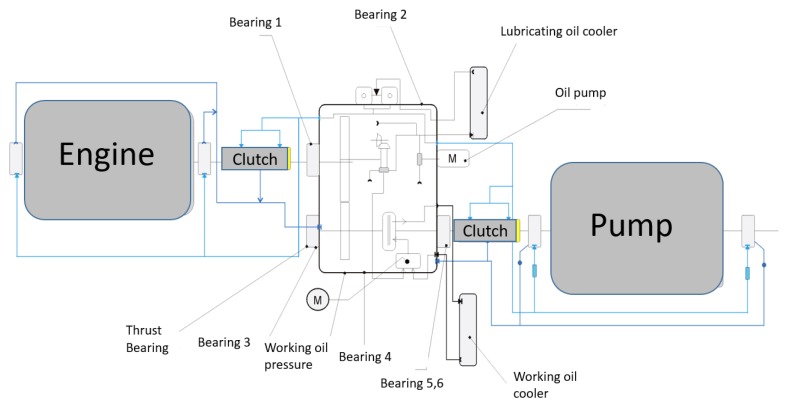
Schema of three stage pump unit.

Feed pumps are designed to supply steam boilers of power units with high power output. The pump consists of three basic units, shown in Figure 1:

1. a feed pump type HD 150 × 8,
2. hydrokinetic coupling,
3. and electric engine.

**Figure 2:**
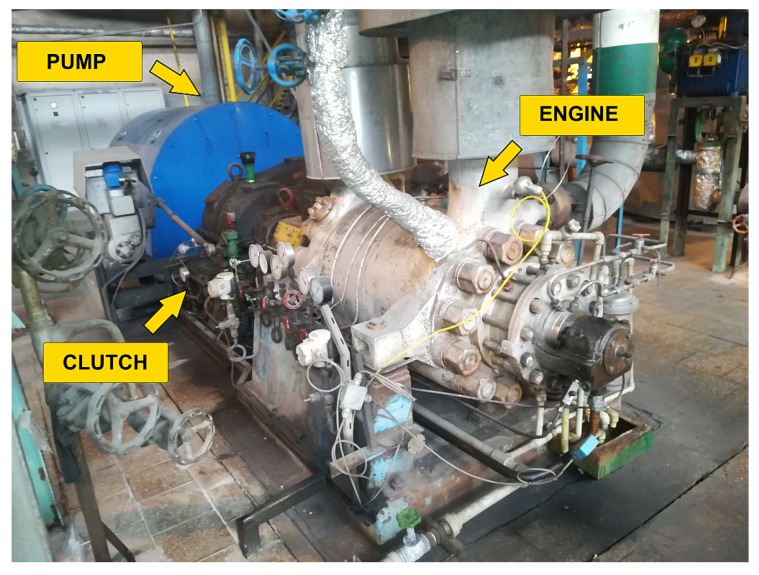
Picture of three stage pump unit with the measuring apparatus.

One steam boiler is powered by three pump units. In order to ensure operation under nominal conditions, two pumps must run in parallel. Usually, one of the pumps is put into reserve and is switched in the event of failure of one of the operating pumps. In the case of failure free operation, the operating schedule ensures a balanced load on all pumps. The pump unit is exposed to typical bearing failures, water/oil leaks, and electrical faults [11,12]. To prevent failures, the pump is regularly checked during day-to-day inspections and subjected to diagnostic reviews over a longer time horizon. Most of the inspection work consists of reading and interpreting the measurements from the measuring apparatus visible in Figure 2.

In this paper, we present a method that allows for early detection of faults in boiler feed pumps at power plants on the basis of signals captured with the use of various sensors mounted on water pumps. For this purpose, we use a bag of regression models built for the particular signals being monitored. The regression models enable the detection of deviations from the operation state without the necessity of labeling data. Therefore, our approach minimizes the requirement of using expert knowledge, in particular the knowledge related to the categorization of failures and the exact time of their occurrence, which is sometimes difficult to identify.

## 2. Related Works

Depending on the impact of potential failure on the production process, different maintenance approaches are applied [13,14,15]. Regardless of whether we apply a reactive, preventive, or predictive maintenance strategy, the main goal is to provide the required capacity for production at the lowest cost [16]. It can be obtained by incorporating machine learning techniques to minimize energy consumption as proposed in [17]. Classic condition monitoring techniques are based on inspections and observation of the physical properties of the device; the techniques used include visual monitoring (contaminant, leaks, thermograph), audible monitoring, and physical monitoring (temperature, vibration) [18,19]. With the real-time analysis of production data and advanced data exploration, we can implement remote condition monitoring and predictive maintenance tools to detect the first symptoms of failure long before the appearance of the first alarms preceding failures of the equipment in a short period [20,21,22]. We can also predict the remaining useful life of components in mechanical transmission systems, e.g., by applying deep learning as one of the most advanced data driven methods, like the new type of long short term memory neural network with macro-micro attention [4]. Many applications are being developed in the field of renewable energy sources. The authors presented in their articles [23,24,25,26] applications for early detection of faults based on SCADA data. By using SCADA data for performance monitoring, for example, it is possible to detect gearbox planetary stage faults early based on gearbox oil temperature rise, power output, and rotational speed [27].

The task is supported by statistical analysis that provides tools for trend analysis, feature extraction, presentation, and understanding of data. On the other hand, various machine learning techniques have been are developed that allow for the automatic creation of complex models based on large datasets. Machine learning based algorithms generally can be divided into two main classes:

- supervised, where information on the occurrence of failures is present in the training dataset;
- unsupervised, where process information is available, but no maintenance related data exist.

Supervised approaches require the availability of a dataset $S={\left\{x_{i},y_{i}\right\}}_{i=1}^{n},$ where a couple $\left\{x_{i},y_{i}\right\}$ contains the information related to the *i*th process iteration. Vector $x_{i}\in R^{1\times p}$ contains information related to the *p* variables associated with available process information [28]. Depending on the type of *y*, we distinguish:

- classification models, if categorical labels are predicted;
- regression models, if the results are continuous values.

Classification and regression may need to be preceded by relevance analysis, which attempts to identify attributes that are significantly relevant to the classification and regression process [29].

Supervised learning is successfully used in the area of predictive maintenance to classify faults by building fault detectors. In the literature, these detectors rely on various Artificial Intelligence (AI) techniques, such as artificial neural networks [30,31], k-nearest neighbors [32], support vector machines [33,34], or Bayesian networks [35,36], frequently using some methods for reducing the dimensionality of the data, such as principle component analysis [37,38].

Unsupervised learning techniques mostly work on the basis of outlier detection algorithms. Outliers may be detected using statistical tests that assume a distribution or probability model for the data or using distance measures where objects that are remote from any other cluster are considered outliers [29]. While choosing the clustering algorithm, it is worth remembering the possibility of applying approximated versions of the algorithms (e.g., the modification of the K-means clustering algorithm described in [39]), which could provide benefits in terms of computation, communication, and energy consumption, while maintaining high levels of accuracy. Building models that do not require labeled data is possible thanks to the use of techniques, such as auto-encoders [40,41], deep belief networks [42], or statistical analysis [43,44].

## 3. Anomaly Detection System

**Figure 3:**
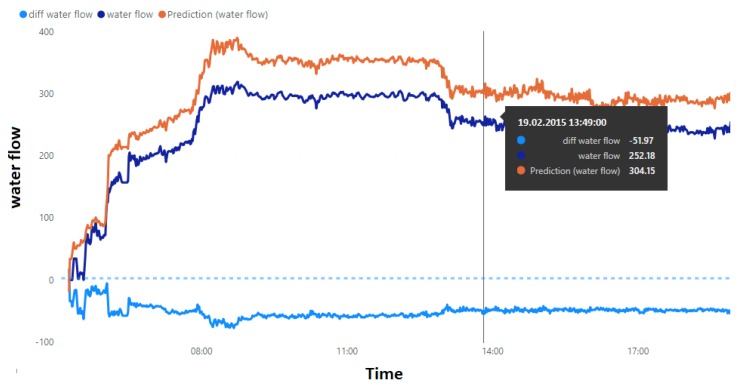
Difference between the real and estimated value of the water flow behind the pump.

Due to uncertain data and limited access to expert knowledge, we decided to develop the anomaly detection system without using classification techniques (i.e., we used the regression models). Therefore, we excluded the information on the performed maintenance works from the learning process. The concept of our anomaly detection system assumed the creation of a model for each of the measurement signals connected with the device and to analyze the differences between the real (measured) and expected values of the signal. The expected value was calculated from the current indications of the other sensors, as in the concept of full signal reconstruction in the method of normal behavior modeling [22]. Our model was trained with historical data from the period preceding the examined time. We assumed that in the time preceding the registered fault, the difference between these values would increase. A sample graph of the estimated and real values of water flow behind the pump is shown in Figure 3, and the graph shows the period within which the minimum flow valve remained in a state of failure; hence, a large prediction error was visible.

The input dataset used in the training phase of the created regression models contained raw historical measurements obtained offline from the Power Generation Information Manager system (PGIM) [45]. The PGIM system was a data repository for signals from the Distributed Control System (DCS) used in power plants. Data included temperatures from various sensors located on the monitored pump unit (e.g., from bearings), oil pressures, electric current, and settings of the operational parameters. The description of all signals is presented in Table 1. The scope of data was from January 2013 to August 2017 with a sampling period of one minute. From this period, we were able to obtain a broad spectrum of reliable and high quality measurements; particular sensors were mounted and constantly monitored, so we were able deduce from the data accurately. In this period, we were also able to identify several unit failures and take appropriate actions, including the development of the presented approach, to avoid them in a subsequent period.

### 3.1. Description of the Input Data

Event data covered failures, operational work, and repairs recorded both in the operator’s logbooks and ERP system. The operator logbook contained manually entered information about the failure of the device and the date of occurrence. Within the period we investigated, two serious defects were recorded, which were the reason for the exclusion of whole units from the normal work. More than 70 events were recorded in relation to other minor defects, detected leaks, planned inspections, etc. All events were categorized as:

- cooler malfunction, 15 incidents,
- cleaning the oil filter, 15 incidents,
- defects of the bearings, 2 incidents,
- oil leaks, 19 incidents,
- water leaks, 8 incidents.

### 3.2. Tools and Methods

**Figure 4:**
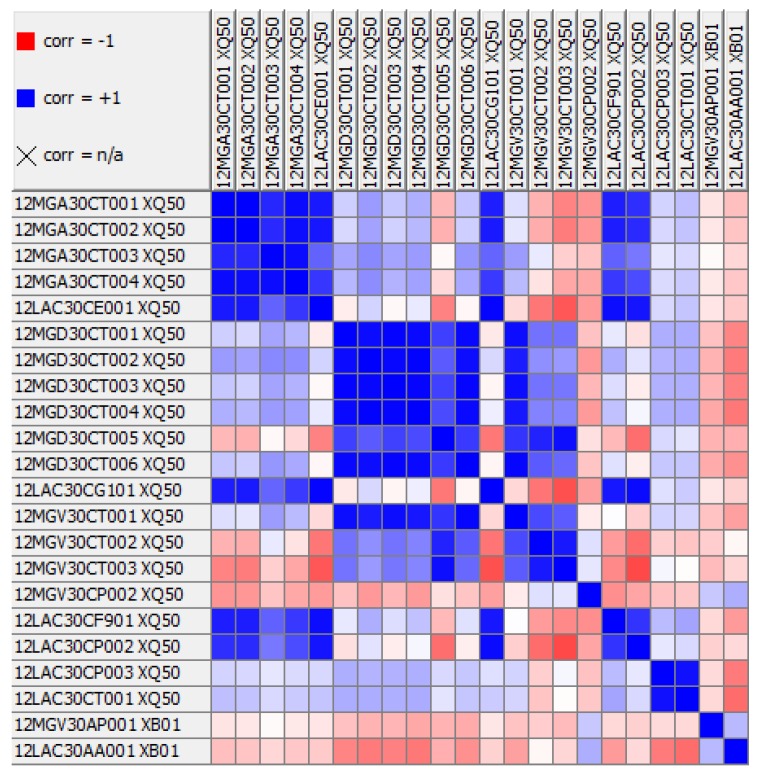
Correlation (corr) matrix for the training set.

The algorithm for detecting anomalies in the boiler feed pump was implemented in the KNIME environment [46]. KNIME is an analytical platform for the graphical design of data analytics workflows. It contains components for data processing, machine learning, pattern recognition, and visualization. To create digital models of the pump, we used the polynomial regression method (the degree of the polynomial was a parameter, and the best results were experimentally obtained for a degree equal to one, i.e., linear regression). The unquestionable advantage of algorithms that rely on regression is their computational simplicity. Other factors that decided the appropriateness of the regression models were linear relationships and a high correlation between variables. The Pearson correlation coefficients calculated for each pair of columns are shown in Figure 4. As we can see, each value was highly correlated with at least one other measurement.

For the set of all input data, where $x=[x_{1},x_{2},x_{3},\ldots ,x_{m}]$ is the vector of individual measurements from *m* sensors, we estimated *k* response variables as a linear function of all available variables, i.e.,

(1) $$x_{i}=\sum_{\begin{array}{c} j=1\\ j\neq i \end{array}}^{m}a_{j}^{\left(i\right)}x_{j}+a_{0}^{\left(i\right)}+\varepsilon^{\left(i\right)}\;\;\forall i\in \left\{1,2,\ldots ,k\right\},\;k\leq m,$$

where $\varepsilon^{\left(i\right)}$ is the *i*th independent identically distributed normal error and coefficients $a_{j}^{\left(i\right)}$ are calculated using the method of least squares [47].

The training dataset for the regression model created was the specific historical time window. The result set contained the response of the model for a current timestamp or, in a more extensive form, it was the time window containing recent results to enable the trend and average values to be analyzed. The size of the training set affected the characteristics of the results. The larger the set was, the smaller the approximation error achieved. The training set should be large enough to detect events spread over time, for example gradual degradation of bearing or increasing leakage. On the other hand, the tmodel should be able to produce reliable results in situations after major overhauls where the training set was not too extensive and the machine may change its performance characteristics.

### 3.3. Algorithm for Compound Predictive Maintenance

**Figure 5:**
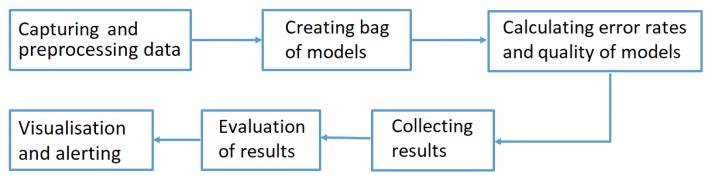
Algorithm flow chart.

The prepared predictive maintenance algorithm was a compound process performed for each monitored signal separately. The diagram shown in Figure 5 illustrates the general algorithm performed while building the compound predictive maintenance model. Its particular steps will be described in the following subsections. The proposed algorithm did not make use of a classical model of multiple regression, but a bag of regression models was created and normalized relative errors calculated for each of the models. Taking into account the maximum error and determining different alert thresholds, faults could be detected much earlier due to the observed abnormality (we could observe significant deviations of the value of a signal from an expected one preceding the recorded failures). It should be stressed that the authors assumed that the regression model was updated every constant time quantum (ΔT), i.e., the dataset was divided into equal parts (covering a fixed period). The data from the previous time period were treated as the dataset for regression function estimations, which were used in the next time period.

#### 3.3.1. Capturing and Preprocessing Data

In the first step, a set of data was loaded into the workflow. In the cleaning process, rows with missing values were fixed. The columns with the timestamp were converted from text to date format. The data were labeled according to whether a device was in operation or out of operation, and then, only the period of operation was considered. The operating threshold was the pump’s power supply value of 1 A, which was the result of disturbances caused by the self-induction of electricity when the pump was switched off. The threshold value was determined as twice the absolute peak value of the self-induced current:

(2) $$\tau_{1}>1A>2\times |peakvalue|,$$

where $\tau_{1}$ is the operating threshold of the pump’s power supply value.

#### 3.3.2. Creating the Bag of Models

In the process of building the prediction data model (presented in Algorithm 1), we considered a set of *k* signals that we wanted to investigate $\left(x_{1},\ldots ,x_{k}\right)$, where k≤m (*m* is the number of all sensors). For each of these signals, we created a regression model based on the remaining variables (according to Equation (1)) and then saved it in the Predictive Model Markup Language (PMML) format. The parameters of the model were the current timestamp $T_{0}$, the length of the time window of the training set ΔT, the collection of *k* selected signals, the threshold for operating state determination $\tau_{1}$, the threshold for the coefficient of determination $\tau_{2}$, and the maximum polynomial degree deg for the regression model built.

**Algorithm 1:** Computing the bag of regression models.

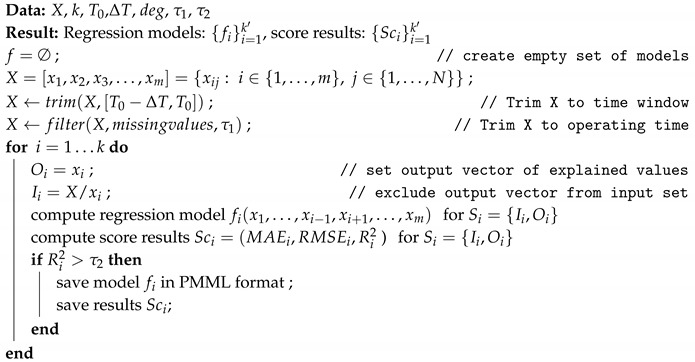



It is worth noting that despite the fact that we had data taken from all *m* sensors, we estimated only k≤m variables because there was no point in determining the regression function for variables that only accepted two values: one (true) or zero (false). However, the true/false values may give us additional information, and that was why we did not omit these variables in determining the regression functions. Furthermore, since we did not save all *k* investigated models, we obtained only $k^{\prime }\leq k$ regression models as a result of our algorithm.

For all i∈{1,…,k} of the computed regression models $f_{i}$, we also computed score results $Sc_{i}$ that included the values of the mean absolute error ($MAE_{i}$), the root mean squared error ($RMSE_{i}$), and the coefficient of determination ($R_{i}^{2}$), which were calculated according to Equation (3). Comparing the value of the coefficient of determination $R_{i}^{2}$ with the threshold value of $\tau_{2}$ allowed us to decide whether to accept or reject the created regression model.

#### 3.3.3. Calculation of Error Rates and Evaluation of the Quality of Models

In order to assess the quality of the *i*th model, we calculated several coefficients, which allowed measuring the differences between the observed and estimated values, i.e., the Mean Absolute Error (MAE), the Root Mean Squared Error (RMSE), and the coefficient of determination $\left(R^{2}\right)$:

(3) $$\begin{array}{ccc} MAE_{i} & = & \frac{1}{n}\sum_{j=1}^{n}\left|x_{ij}-{\hat{x}}_{ij}\right|,\\ RMSE_{i} & = & \sqrt[{2}]{\frac{1}{n}\sum_{j=1}^{n}{\left(x_{ij}-{\hat{x}}_{ij}\right)}^{2}},\\ R_{i}^{2} & = & 1-\frac{\sum_{j=1}^{n}{\left(x_{ij}-{\hat{x}}_{ij}\right)}^{2}}{\sum_{j=1}^{n}{\left(x_{ij}-\bar{x_{i}}\right)}^{2}}, \end{array}$$

where $x_{i1},\ldots ,x_{in}$ are the observed values, ${\hat{x}}_{i1},\ldots ,{\hat{x}}_{in}$ the predicted values, $\bar{x_{i}}$ the average value for the *i*th variable (estimation for the *i*th sensor for i∈{1,2,…,k}), and *n* the number of analyzed samples in the time window ΔT.

#### 3.3.4. Collecting Results and Evaluation

The difference between the observed and the predicted values was not an absolute measure that could be used when comparing the values with other signals. In order to normalize the results, we introduced the NRE (normalized relative error) coefficient (which was a multiple of the mean standard deviation) to measure the degree of deviation for the *i*th variable in a dataset ($i\in \{1,2,\ldots ,k^{\prime }\}$).

(4) $$NRE_{i}=\frac{|x_{i}\left(t\right)-{\hat{x}}_{i}\left(t\right)|-MAE_{i}}{RMSE_{i}},$$

where $x_{i}\left(t\right)$ and ${\hat{x}}_{i}\left(t\right)$ are the current value and its estimation obtained by using the regression function ($t\in (T_{0},T_{0}+\Delta T)$).

By selecting a variable with the maximum value of the $NRE_{max}$ (called the maximum normalized relative error), we could identify the signal that was probably the cause of the upcoming fault:

(5) $$NRE_{max}=\max\left(NRE_{1},NRE_{2},\ldots ,NRE_{k^{\prime }}\right).$$

This made it possible to diagnose a source of the anomaly more quickly.

#### 3.3.5. Visualization and Alert Triggering

To visualize the results of the created model, we used the PowerBI software, which allows for the easy and fast presentation of data, including time series. The warning threshold (first level alert), proportional to the RMSE value, was set to three:

(6) $$NRE_{max}>3\Rightarrow 1st\;level\;alert,$$

and the failure (second level alert) was signaled if $NRE_{max}$ reached the value of six:

(7) $$NRE_{max}>6\Rightarrow 2nd\;level\;alert.$$

The value of the warning threshold was set to three because if our model was ideal and our error was normally distributed N(0,1), the three-sigma rule would state that 99.73% of the values lied within the three-sigma interval. However, even for non-normally distributed variables, according to Chebyshev’s inequality, at least 88.8% of cases should fall within properly calculated three-sigma intervals.

The detected anomalies in various signals leading to final failures are presented in Section 4.

## 4. Results

### 4.1. Model Accuracy and Parameter Optimization

In order to ensure the satisfactory quality of the model and to evaluate it, a number of experiments were carried out to select appropriate parameters such as a polynomial degree or time window length. The experiments were carried out on the same test set, and the measures of model accuracy were the coefficient of determination $\left(R^{2}\right)$ and the average percentage value of Mean Absolute Error (%MAE).

**Figure 6:**
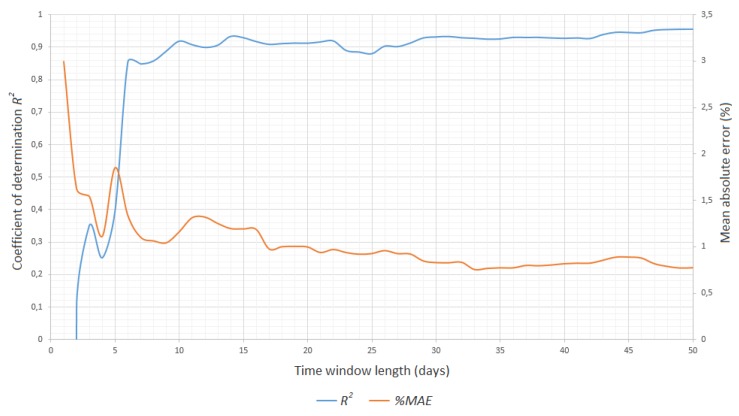
Mean absolute error in the time window length function.

The length of the time interval had a direct impact on the number of calculations in the model. In the Industrial IoT, delays and the scalability of the system are important, so heuristics should be considered in order to simplify the calculations to achieve the expected result without overloading resources [39]. The results shown in Figure 6 represent the percentage mean value of the mean absolute error in relation to the length of the training dataset expressed in days (one day is 1440 rows). The graph shows that the bigger the number of samples, the smaller the error, but the satisfactory quality was obtained after just one week of work. After 20 days, the level of %MAE and $R^{2}$ stabilized. The time window of 30 days that we chose was a tradeoff between stable values of %MAE and $R^{2}$ and the learning time. This had a significant impact on the ability to fit the model within a short period after major repairs or a longer downtime.

Table 2 shows the results obtained depending on the regression method used. The results of the algorithms were comparable, and the best fit was achieved for linear regression. In the case of increasing the polynomial degree of polynomial regression, we observed a decrease in model accuracy.

Detailed results obtained for each of the modeled signals for the linear regression algorithm are shown in Table 3.

### 4.2. Observation of Deviations in the Control Chart

**Figure 7:**
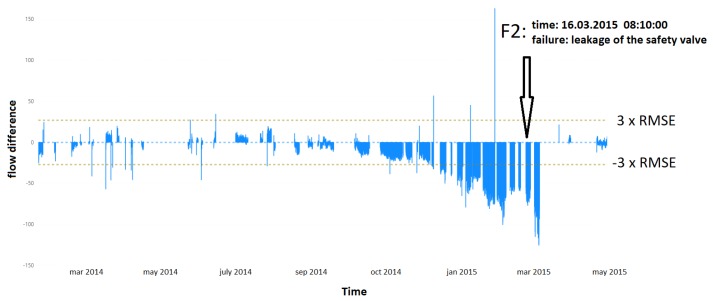
Difference between the real and estimated value of the water flow. F, Failure.

**Figure 8:**
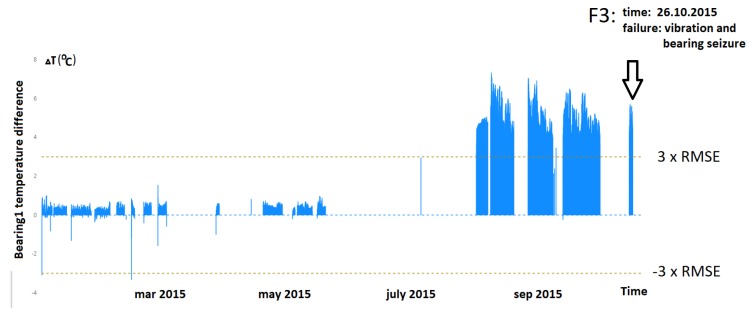
Difference between the real and estimated value of temperature of Bearing 1.

**Figure 9:**
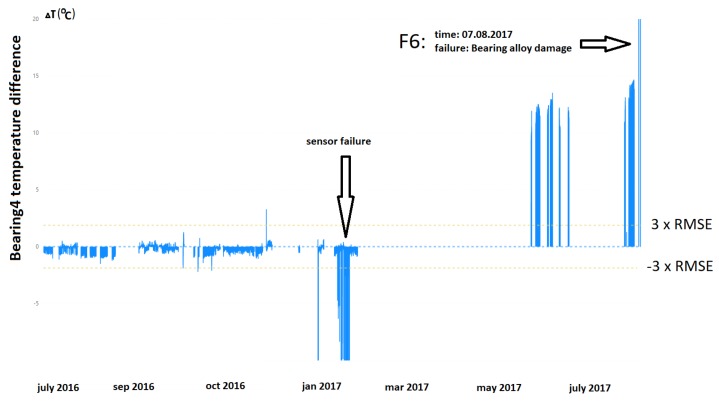
Difference between the real and estimated value of temperature of Bearing 4.

Analyzing the differences between the actual value and the estimated value, we could observe significant deviations preceding the recorded failures. In a few examples (Figure 7, Figure 8 and Figure 9), we analyzed the results obtained against the background of significant failures. In the first example (Failure F2) shown in Figure 7, we observed a gradual decrease in pump performance due to leakage of the relief valve. The relief valve is used to protect the pump against seizure during start-up and low speed operation. The fault was recorded on 16 March 2015. By determining the alert threshold as three standard deviations (3×RMSE), we could have detected this event three months earlier.

**Figure 10:**
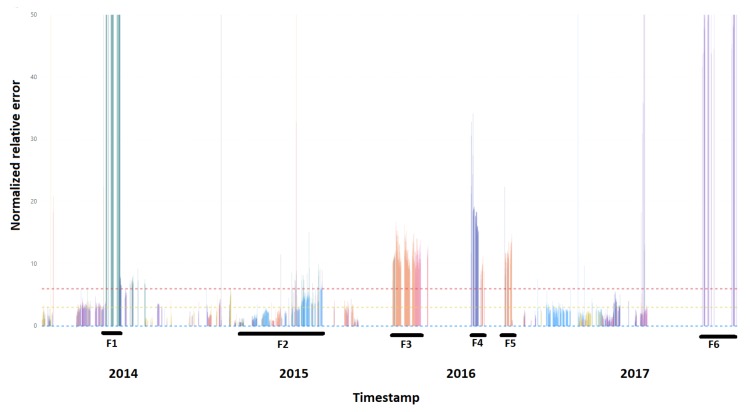
Normalized relative error (NRE) with colored sources of events and failures that occurred.

The second example (Failure F3) showed a sudden increase in the deviation of the temperature of the bearing. The anomaly occurred after a period of equipment downtime. The reasons for the failure were the lack of concentricity of the gear and motor shafts and the melting of the bearing alloy. The visible consequences of the failure were vibration and smoke from the bearing. Differences in the temperature leading to this failure are shown in Figure 8. Failure events marked with symbols F4 and F5 in Figure 10 were most probably the result of degradation and contamination caused by the fault F3.

The third example (F6) and the second critical failure visible in Figure 9 showed damage to a bearing alloy that resulted in an elevated bearing temperature. As in the previous example, a significant change in the system operation characteristics was observed after a longer downtime; in this case, a growing trend of the deviation curve could be observed a few days before the failure.

### 4.3. Results and Visualization

The presentation of the results does not focus on the detection of a specific event, but indicates the specific measurement for which there was the greatest deviation, being the symptom of a potential upcoming fault. The proposed method allowed for a preliminary interpretation and prioritization of the results before the process of further data drilling. The results of the algorithm for a three month moving window for the whole time series are presented in Figure 10. With F1–F6, we mark the detected (predicted) failures of equipment that really occurred in the monitoring period. Two vertical lines represent the first and the second alert level, as described in Formulas (6) and (7). All detected failures are described in more detail in Table 4. The color of the line on the chart indicates the signal for which the greatest relative deviation occurred. The error rate was calculated as shown in Equation (4).

The algorithm used was very sensitive to events related to sensor failure. The failure of one of the sensors had a significant impact on the results obtained from the regression models for signals gathered from the whole device. Disturbed signal had a negative impact on the quality of the results both due to the disturbance of the training process and the presentation of the results. In the case of visualization, bad measurement made the presentation and interpretation of the results improper. However, during the training process, measurement error caused the calculation of lower weights of coefficients, which had a significant impact on the quality of the model. Therefore, in order to improve the quality of the model, data for which incorrect measurements occurred should be excluded in the process of data cleaning.

To compare the results with other algorithms described in related work, we created models based on decision trees and multi-layer perceptron (MLP) classification algorithms. In both cases, we performed the experiments by providing raw data and adding a step of feature extraction and dimensionality reduction using the PCA algorithm. Feature extraction consisted of calculating additional variables for each input signal using functions such as kurtosis, skewness, standard deviation, minimum, maximum, variance, and mean. Labels were assigned to the dataset informing about the occurrence of a failure for each sample. The experiments were conducted using the cross-validation technique. The dataset was divided into 10 equal parts, and calculations were performed in a loop where one part was a test set and the remaining nine parts were a training set.

The results of the experiment containing statistics such as accuracy, the area under the curve, sensitivity, and specificity are shown in Table 5.

**Figure 11:**
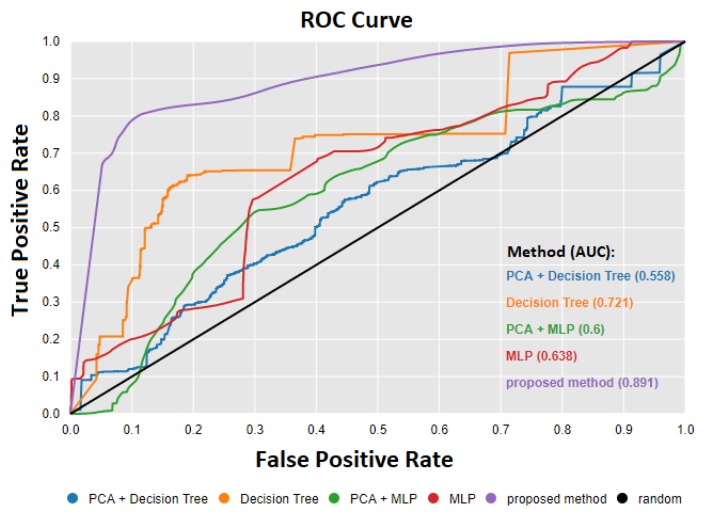
Receiver operating characteristic.

Receiver Operating Characteristic curves (ROC) shown in Figure 11 visualize the performance of the proposed model compared to the others. The quality of the algorithm was determined by the AUC (Area Under Curve) value. The AUC took values between zero and one, where one was the optimum result.

## 5. Discussion

The creation of specifically designed algorithms for predicting failures in the energy sector, like the one presented in this paper, is important for several reasons. First of all, they are essential elements of predictive maintenance processes performed at real power plants in Poland (where we performed our research), but can also be used in other countries. Secondly, early prediction of serious failures, which is possible owing to the presented method, prevents many people and various factories from being cut off from electricity. As a consequence, which is the third important reason at the same time, this helps to avoid significant economic losses not only for the power plant itself, but also for production companies cut off from electricity in the event of a failure.

Although the presented method belongs to the group of supervised algorithms, its use does not require much analytical skill and effort because the label in the learning process was, in fact, an element of the input dataset. Therefore, without expert knowledge, detailed analysis of historical events, and knowledge of production processes, we could use this method quickly and efficiently in many areas. Many algorithms for detecting anomalies are dedicated to single stream data [48]. However, in the case of the method presented in the paper, signals were characterized by unforeseen variability depending on control signals and, at the same time, high inertia (e.g., temperature). When considering the application of PCA based methods [37,49], we could face the problem that the device operates in different states. The pump operates in different load ranges, variable atmospheric conditions, and with different types of materials used (e.g., oil, grease), so that the abnormal states reflected in small deviations from predicted values are not easily visible in the multidimensional space of PCA coefficients. Moreover, the reduction of dimensionality causes the blurring of small deviations between the correlated signals, making it impossible to detect abnormalities, which is the essence of the algorithm proposed in this article.

The presented approach complemented existing approaches for failure prediction in several dimensions:

- It allowed predicting failures of a particular unit (i.e., water pump) at a power plant not on the basis of the main variable describing the process being performed (water flow in our case), but on correlated signals from other sensors located on the monitored unit.
- In contrast to methods that rely on classification (e.g., [28,50,51]), it did not require labeling datasets that consisted of the training data, so an expert was required only to assess the convergence of the results returned by the model with the actual breakdowns and, possibly, to initiate corrections (i.e., change the alert thresholds, change the length of the training set).
- While comparing our approach to PCA based methods known from the literature, we noticed that the reduction of dimensionality caused failure and correct states to become indistinguishable. Therefore, with the use of such solutions, we were not able to assess correctly that a failure would occur and when we should take appropriate corrective actions.

The overall advantages and disadvantages of the different predictive maintenance application techniques are shown in Table 6.

Portability was one of the properties of the presented solution. This portability simplified the implementation of the algorithms in various environments. Since the presented approach relied on building a bag of models that were exported to the PMML (Predictive Model Markup Language), it allowed moving ML models from one production environment to another. So far, driven by the research conducted at one of the power plants managed by the TAURON company in Poland, the algorithms were developed and tested offline on the basis of historical data gained from SCADA systems. However, the use of the PMML format enabled the implementation of algorithms in the large monitoring IT infrastructure. Due to the scaling capabilities, the monitoring infrastructure may be built in one of the cloud platforms, such as Amazon Web Services (AWS) or Microsoft Azure. Both platforms provide rich IoT suites for building monitoring centers that collect (through Azure IoT Hub/Event Hub or AWS IoT Core services), store (e.g., Azure BLOB, AWS S3, CosmosDB, DynamoDB), process (e.g., Azure Stream Analytics, Amazon Kinesis), and analyze (Azure Machine Learning + Azure Functions or AWS SageMaker + AWS Lambda) data from IoT devices, visualize the results of analysis (Power BI or Amazon QuickSight), and finally, notify management staff (Azure Notification Hub or Amazon Simple Notification Service). In the AWS cloud, PMML representations of the created predictive models may be imported through AWS Lambda with JPMML, a PMML producer and consumer library for the Java platform. Such an implementation would allow us to decouple the system into various distributed microservices and scale them separately according to current needs.

The presented algorithm not only could be applied to water pumps, but also to other devices, if only the correlation between the signals in a different operational environment allows for proper signal prediction. In order to adapt the method, the $\tau_{1}$ operating threshold and alert level thresholds should be redefined, and the name of the signal responsible for powering the system should be changed in the algorithm (by applying only these changes, we were able to use the algorithm for monitoring failures of the oxygenation compressor unit).

## 6. Conclusions and Further Works

The observed results confirmed the suitability of the algorithm used to detect anomalies. A significant correlation between the input signals enabled the models to capture abnormal events. However, the method was also very sensitive to any changes in a system being examined, especially in situations when one of the sensors breaks down. On the other hand, the advantage of the algorithm was the possibility to predict serious failures long before their real occurrence and identify the signal that indicated a potential source of the failure event. This allowed detecting the source of the incoming failure. One of the possibilities to optimize the model could be to add a step in the pre-processing of the device state estimation [52,53,54]. Currently, we used rule based techniques to determine whether a device was working or was turned off. In order to limit false positives in the results, a preliminary categorization of the state for each signal, including downtime, start-up, operation, and damage, could be considered. In comparison with other methods presented in the related literature, the advantage of the presented method was its applicability in the case of insufficient data describing the predicted events only on the basis of process data. Due to its simplicity, the algorithm worked very quickly and did not require large hardware expenses. Based on the results obtained, it is possible to improve the algorithm by adding a classification level, where the calculated values of deviations are one of the inputs of the classifier. The algorithm could also be used in the data cleaning and data preparation steps in order to remove incorrect data from the dataset (i.e., measurements from damaged sensors and from periods when the machine was working in the state of failure).

## Figures and Tables

**Table 1:** Description of the input data collected by the sensors located on the pump unit.

Signal Name	Unit	Min	Max	Avg	Description
12MGA30CT001 XQ50	°C	0	150	45.0	temperature of the motor stator windings
12MGA30CT002 XQ50	°C	0	150	45.2	engine iron temperature
12MGA30CT003 XQ50	°C	0	150	34.8	engine cooling air temperature
12MGA30CT004 XQ50	°C	0	150	47.9	air temperature behind the engine
12LAC30CE001 XQ50	A	0	400	217	motor power supply current
12MGD30CT001 XQ50	°C	0	100	54.9	Bearing No. 1 temperature
12MGD30CT002 XQ50	°C	0	100	57.9	Bearing No. 2 temperature
12MGD30CT003 XQ50	°C	0	100	58.4	Bearing No. 3 temperature
12MGD30CT004 XQ50	°C	0	100	60.7	Bearing No. 4 temperature
12MGD30CT005 XQ50	°C	0	100	69.3	Bearing Nos. 5. 6 temperature
12MGD30CT006 XQ50	°C	0	100	56.2	thrust bearing temperature
12LAC30CG101 XQ50	%	0	100	53.4	clutch attitude
12MGV30CT001 XQ50	°C	0	100	81.1	lubricating oil temperature in front of the cooler
12MGV30CT002 XQ50	°C	0	100	84.3	working oil temperature behind the cooler
12MGV30CT003 XQ50	°C	0	150	111	working oil temperature in front of the cooler
12MGV30CP002 XQ50	MPa	0	1	0.56	lubricating oil pressure
12LAC30CF901 XQ50	t/h	0	450	246	water flow
12LAC30CP002 XQ50	MPa	0	25	14.0	output water pressure
12LAC30CP003 XQ50	MPa	0	4	0.66	supply water pressure
12LAC30CT001 XQ50	°C	0	400	148	temperature of the discharge nozzle
12MGV30AP001 XB01	True/False	0	1	-	setting the oil pressure
12LAC30AA001 XB01	True/False	0	1	-	setting the minimum flow valve

**Table 2:** Accuracy of regression methods.

Method	MAE	$R^{2}$
decision tree regression	1.17%	0.8683
gradient boosted trees regression	1.95%	0.8725
linear regression	1.04%	0.8818
polynomial regression 2 deg	1.12%	0.7941
polynomial regression 3 deg	1.09%	0.8383
polynomial regression 4 deg	1.24%	0.7069
polynomial regression 5 deg	1.22%	0.4599

**Table 3:** Accuracy of each model for linear regression.

Model	MAE	$R^{2}$
water flow	1.53%	0.9669
lubricating oil pressure	1.10%	0.2429
engine iron temperature	0.08%	0.9975
thrust bearing temperature	2.49%	0.8401
Bearing No. 1 temperature	0.89%	0.9788
Bearing No. 2 temperature	0.48%	0.9914
Bearing No. 3 temperature	1.03%	0.9707
Bearing No. 4 temperature	1.32%	0.9204
Bearing Nos. 5. 6 temperature	1.09%	0.961
lubricating oil temperature in front of the cooler	1.68%	0.817
lubricating oil temperature behind the cooler	0.88%	0.9171
air temperature behind the engine	0.82%	0.8662
temperature of the stator windings	0.15%	0.9936
average	1.04%	0.8818

**Table 4:** Predicted events and time of their recording.

Id	Description	Registration Date	Start of Failure	Source
F1	Defective measurement of lubricating oil temperature in front of the cooler	25.12.2013	27.11.2013	Lubricating oil
F2	Leakage of the relief flow valve	27.02.2015	06.01.2015	Water flow
F3	Vibrations and smoke from the internal bearing of the pump drive motor	14.01.2016	26.10.2015	Bearing 1
F4	Oil leakage from the shaft from the outer bearing of the engine	03.02.2016	18.01.2016	Lubricating oil
F5	Poor cooling water flow through the cooler	08.04.2016	30.03.2016	Bearing 1
F6	Increased temperature of Bearing No. 4 of the VOITH gearbox	07.08.2017	22.05.2017	Bearing 4

**Table 5:** Accuracy statistics.

	Accuracy	AUC	Sensitivity	Specificity
Proposed method	0.86	0.89	0.67	0.95
Decision tree	0.76	0.72	0.58	0.85
PCA + Decision tree	0.59	0.56	0.43	0.67
MLP	0.67	0.64	0.2	0.90
PCA + MLP	0.65	0.60	0.37	0.79

**Table 6:** Benefits and limitations of different approaches for the considered problem.

	Proposed Method	Classification (e.g., Decision Trees, SVM)	Anomaly Detection (e.g., Deep Neural Networks, Autoencoders, PCA)
Fault detection capability	Possible detection of failures in the short and medium term	Possibility to detect for a certain period before failure or to determine the expected time to failure (remaining useful life)	Possible by detecting outliers
Ability to diagnose faults	Possibility to determine the source signal of the deviation	Possibility of classifying failures; however, the limitation is that the predicted events must be known in the training set	Algorithms of this type are not able to classify failures
The need for data labeling	NO	YES	NO
Expertise needed	Interpretation of results, setting alarm thresholds	The extensive expert knowledge needed and knowledge of the processes being modeled	Interpretation of results, process knowledge required
Training speed	Very fast	Slow	Average

## Abbreviations

The following abbreviations are used in this manuscript:

AI	Artificial Intelligence
ANN	Artificial Neural Networks
API	Application Programming Interface
AUC	Area Under Curve
AWS	Amazon Web Services
BN	Bayesian networks
CSV	Comma Separated Values
DCS	Distributed Control System
ERP	Enterprise Resource Planning
IoT	Internet of Things
kNN	k-Nearest Neighbors
MAE	Mean Absolute Error
MLP	Multi Layer Perceptron
NRE	Normalized Relative Error
PCA	Principle Component Analysis
PGIM	Power Generation Information Manager
PMML	Predictive Model Markup Language
RMSE	Root Mean Squared Error
ROC	Receiver Operating Characteristic
SCADA	Supervisory Control And Data Acquisition
SDK	Software Development Kit
SVM	Support Vector Machines
